# Hair Growth Regulation by Fibroblast Growth Factor 12 (FGF12)

**DOI:** 10.3390/ijms23169467

**Published:** 2022-08-22

**Authors:** Jiwon Woo, Wonhee Suh, Jong-Hyuk Sung

**Affiliations:** 1 College of Pharmacy, Yonsei Institute of Pharmaceutical Sciences, Yonsei University, Incheon 21983, Korea; wooji001228@gmail.com; 2 Department of Global Innovative Drug, The Graduate School of Chung-Ang University, Seoul 06974, Korea; 3 Epi Biotech Co., Ltd., Incheon 21983, Korea

**Keywords:** fibroblast growth factor 12, hair growth, anagen induction, outer root sheath cell, cell migration

## Abstract

The fibroblast growth factor (FGF) family has various biological functions, including cell growth, tissue regeneration, embryonic development, metabolism, and angiogenesis. In the case of hair growth, several members of the FGF family, such as FGF1 and FGF2, are involved in hair growth, while FGF5 has the opposite effect. In this study, the regulation of the hair growth cycle by FGF12 was investigated. To observe its effect, the expression of FGF12 was downregulated in mice and outer root sheath (ORS) by siRNA transfection, while FGF12 overexpression was carried out using FGF12 adenovirus. For the results, FGF12 was primarily expressed in ORS cells with a high expression during the anagen phase of hair follicles. Knockdown of FGF12 delayed telogen-to-anagen transition in mice and decreased the hair length in vibrissae hair follicles. It also inhibited the proliferation and migration of ORS cells. On the contrary, FGF12 overexpression increased the migration of ORS cells. FGF12-overexpressed ORS cells induced the telogen-to-anagen transition in the animal model. In addition, FGF12 overexpression regulated the expression of PDGF-CC, MDK, and HB-EGF, and treatment of these factors exhibited hair growth promotion. Altogether, FGF12 promoted hair growth by inducing the anagen phase of hair follicles, suggesting the potential for hair loss therapy.

## 1. Introduction

During the hair growth cycle, there are three major phases, known as anagen (growth) phase, catagen (regression) phase, and telogen (resting) phase [1]. During the anagen, epithelial stem cells are stimulated to proliferate and migrate downwards and upwards forming a mature hair follicle [2]. Those undifferentiated epithelial stem/progenitor cells are known to reside in the bulge and outer root sheath (ORS) region [3]. Those cells are usually stimulated by the interaction with the specialized mesenchymal cells, called dermal papilla (DP) [4]. After anagen, the proliferation and migration rate of matrix and ORS cells slow down and the apoptosis of hair cells, including ORS cells, occur [5,6]. Then, the follicle starts to regress, entering the catagen phase [7]. Through catagen, the hair follicle shrinks and enters the telogen phase where the hair shaft stops growing and then eventually detaches from the hair follicle [8,9]. Altogether, these phases of the hair growth cycle are repeated during the course of our lives [10].

The fibroblast growth factor (FGF) family is a significant growth factor that has been widely studied for its biological function including cell growth, tissue regeneration, embryonic development, metabolism, and angiogenesis [11,12,13,14,15]. Well-known biological functions of FGFs have been employed to regenerate damaged tissues such as skin, blood vessels, muscle, adipose, bone, and nerve [16,17,18,19,20]. Of interest, many previous studies demonstrated the role of FGFs in hair growth. The studies shown in Table 1 suggested that other unstudied FGF members might also be involved in the regulation of the hair growth cycle.

FGF12 lacks the N-terminal signal sequence, unlike most FGF family members. Instead, it contains basic residue clusters which allow FGF12 to have nuclear localization signal. FGF12 does not bind to any of the seven major FGF receptors on the cell surface and when FGF12 is transfected into mammalian cells, the protein accumulates in the nucleus and is not secreted [42,43]. Intriguingly, FGF12 is known to have a similar structure to FGF1, which stimulates cell proliferation and hair growth [44]. However, the function of FGF12 has only been studied in nervous and cardiovascular systems and its involvement in the regulation of the hair growth cycle has not been reported [45,46]. In addition, a previous study measured the expression of FGF12 in the mouse skin during various phases of the hair cycle. The expression of FGF12 increased during early anagen, suggesting that it might be involved in hair growth [47]. Altogether, given that various FGF family members have shown their effects on the hair growth cycle, this research investigated the promoting effect of FGF12 on the hair growth cycle.

## 2. Results

### 2.1. Expression of FGF12 during the Hair Growth Cycle

**Figure 1:**
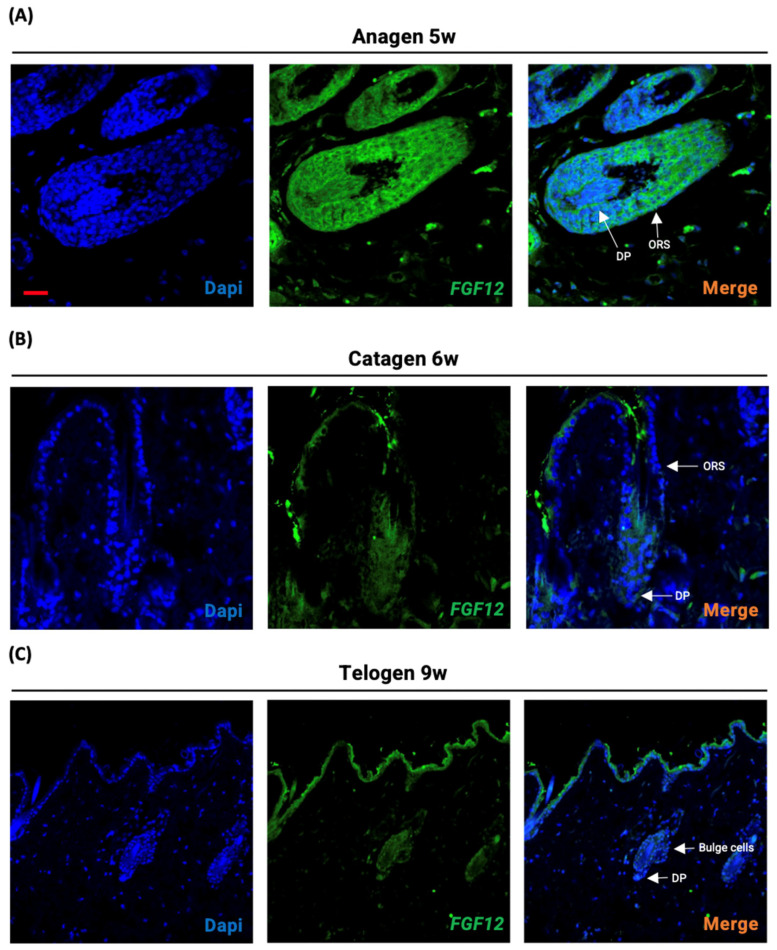
Localization of FGF12 during the hair growth cycle. (**A**–**C**) Immunofluorescence images show expression of FGF12 protein in hair follicles of C3H/HeN mouse. Longitudinal sections of dorsal skins stained with anti-FGF12 antibody (Green) and Dapi (Blue) during anagen at 5-week (**A**), catagen at 6-week (**B**), and telogen at 9-week (**C**) phases. scale bar = 50 μm.

The localization of FGF12 was investigated by immunofluorescence staining of the hair follicles. To analyze the expression of FGF12 during the hair growth cycle, the FGF12 expression in anagen, catagen, and telogen hair follicles was compared. The result confirmed the presence of FGF12 in the entire hair follicle region including the hair bulb and outer root sheath (ORS) during the anagen (Figure 1A). However, during the catagen and telogen, the specific location was not able to be detected in the hair follicle as FGF12 expression decreased (Figure 1B,C). This result is consistent with the previous report that the mRNA expression of FGF12 is increased during the anagen phase, while it is very low in the hair regression period [47]. On the contrary, FGF12 expression is relatively high in the skin epidermis during catagen and telogen. The immunofluorescence staining results of anagen hair follicles invite further investigation of the FGF12 function in the hair growth cycle.

### 2.2. FGF12 Is a Necessary Factor in the Hair Growth Cycle

**Figure 2:**
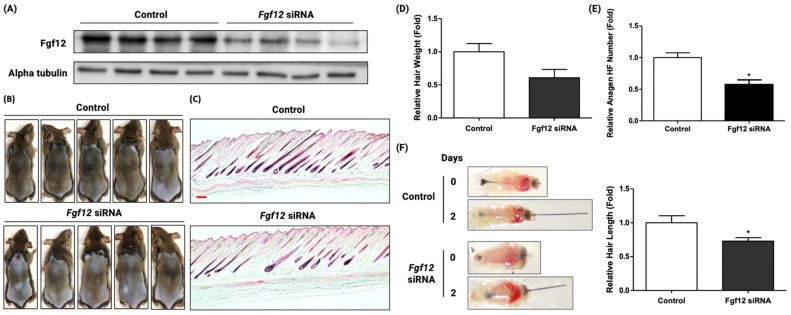
Fgf12 knockdown delayed anagen entry of mouse hair follicles. (**A**) Western blotting confirmed the siRNA-mediated knockdown of Fgf12 in mouse dorsal skins. (**B**) When compared to negative controls, telogen-anagen transition in C3H/HeN mouse was delayed by Fgf12 siRNA injection into the mouse dorsal skin; *n* = 5. (**C**) Hematoxylin and eosin (H&E) staining of the mouse dorsal skin in each group is shown. scale bar = 100 μm. (**D**) The hair weight of each group was measured; *n* = 5. (**E**) Quantification of anagen follicle number in H&E-stained images indicates that Fgf12 siRNA delays anagen entry of hair follicles. (**F**) Mouse vibrissae follicles were isolated and incubated with Fgf12 siRNA. Fgf12 knockdown exhibited decreased hair fiber length compared to the control group; *n* = 10–15, ×1.5 magnification. All data were expressed as mean ± standard error (* *p* < 0.05 versus the control group).

To investigate the effect of Fgf12 in the hair growth cycle, the telogen-anagen transition of mouse dorsal hair follicles was examined by Fgf12 siRNA injection. Firstly, the telogen mouse model was generated by depilating mouse dorsal skin. Depilating dorsal skin of 7-week-old mice immediately allowed hair follicles to undergo a hair cycle in a homogenous manner. After that, Fgf12 siRNA was injected every two days for 14 days. When Fgf12 was knocked down by siRNA in hair follicles, the entry of the anagen phase was delayed (Figure 2B,C). In Fgf12 siRNA injected mouse, the number of anagen hair follicles was lower than the anagen follicles in the control group (Figure 2D,E). The results suggested that Fgf12 is a required factor for the anagen entry. To confirm whether the siRNA knocked down Fgf12 in vivo, proteins of sample tissues from Figure 2B were extracted. By performing Western blot, decreased expression of Fgf12 in mouse dorsal tissue was confirmed at the protein level (Figure 2A). In the mouse vibrissae organ culture, Fgf12 siRNA treatment decelerated the growth of hair follicle fiber length (Figure 2F). Considering the hair growth delayed by siRNA-mediated Fgf12 knockdown, this finding suggested that Fgf12 is a necessary factor for the hair growth cycle.

### 2.3. FGF12 Knockdown in ORS Cells Inhibited the Proliferation and the Migration Rate

**Figure 3:**
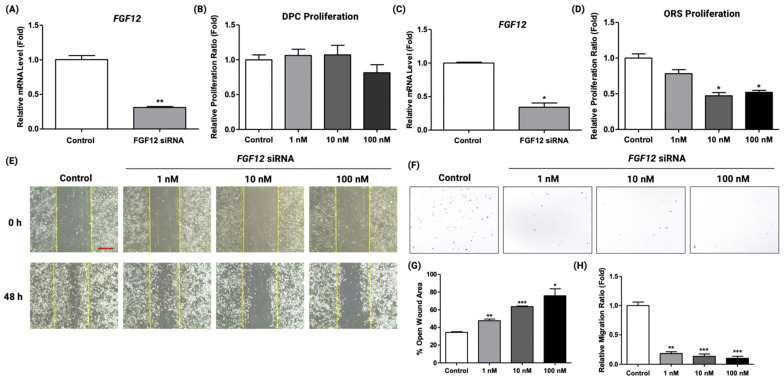
siRNA-mediated knockdown of FGF12 inhibited the proliferation and migration of ORS cells. (**A**) qRT-PCR result confirmed the siRNA-mediated knockdown at mRNA level in dermal papilla (DP) cells; *n* = 3. (**B**) The proliferation property of DP cells was examined with FGF12 siRNA treatment in a dose-dependent manner; *n* = 3. (**C**) qRT-PCR result confirmed the siRNA-mediated knockdown of FGF12 at mRNA level in outer root sheath (ORS) cells; *n* = 3. (**D**) The proliferation property of ORS cells was examined with FGF12 siRNA treatment in a dose-dependent manner; *n* = 3. FGF12 knockdown decreased the proliferation rate of ORS cells at the concentration of 10 nM and 100 nM. (**E**) FGF12 knockdown inhibited the migration of ORS cells in a wound scratch assay. The images at 0 h were taken immediately after the scratch was made. After 48 h, the migration of ORS cells was recorded. scale bar = 50 μm. (**F**) Representative images of transwell migration assay upon treatment of FGF12 siRNA are shown. siRNA-mediated FGF12 knockdown significantly suppressed the migration of ORS cells. (**G**) Wound scratch assay results were quantified and represented as percentages of open wound area; *n* = 3. (**H**) Quantification of transwell migration assay indicated that ORS cell migration was inhibited by siRNA-mediated FGF12 knockdown; *n* = 3. All data expressed as mean ± standard error (* *p* < 0.05, ** *p* < 0.01, *** *p* < 0.001 versus the control group).

Earlier studies revealed that the proliferation and migration of dermal papilla (DP) and ORS cells promote the onset of anagen, which influences the hair growth cycle [3,4]. To further investigate how FGF12 is involved in the hair growth cycle, the effect on the proliferation and the migration properties of DP and ORS cells were studied using FGF12 siRNA. First, the downregulation of FGF12 in DP and ORS cells was confirmed at the mRNA level by qRT-PCR (Figure 3A,C). Afterward, the proliferation assay of DP cells was performed with FGF12 siRNA treatment in a dose-dependent manner (1, 10, and 100 nM). The result suggested that FGF12 knockdown did not affect the proliferation property of DP cells (Figure 3B). On the other hand, the proliferation of ORS cells decreased by around 50% at the concentration of 10 nM and 100 nM compared to the control group. (Figure 3D). Furthermore, to observe the migration property of ORS cells, a wound-scratch migration assay was carried out with the dose-dependent FGF12 siRNA treatment. The result showed that FGF12 knockdown inhibited the migration of ORS cells in a dose-dependent manner (Figure 3E). Compared to the control group, the scratch width was significantly greater in FGF12 knocked down ORS cells (Figure 3H). In the transwell migration assay, migration of ORS cells was quantified using crystal violet staining. The result showed that the siRNA-mediated downregulation of FGF12 inhibited the migration of ORS cells (Figure 3F,G). Altogether, the results revealed that FGF12 is involved in the proliferation and migration of ORS cells during hair growth.

### 2.4. FGF12 Overexpression Promoted the Migration Property of ORS Cells

**Figure 4:**
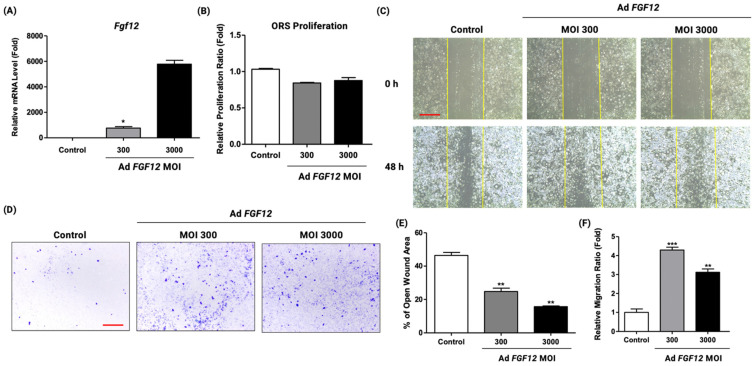
FGF12 overexpression by adenovirus infection promoted the migration of ORS cells. (**A**) Using FGF12 adenovirus, FGF12 was overexpressed in ORS cells at 300, 3000 multiplicities of infection (MOI). Adenovirus (Ad)-infected ORS cells were cultured for 72 h and the mRNA level of FGF12 at 300, 3000 MOI was determined by qRT-PCR. For the control group, Ad-LacZ was infected in ORS cells; *n* = 3. (**B**) The proliferation rate of ORS cells was measured; *n* = 3. Overexpression of FGF12 did not dramatically increase the rate of proliferation. (**C**) Wound-scratch assay was performed with Ad FGF12-infected ORS cells with various MOI. The migration of ORS cells was promoted at 300 and 3000 MOI of adenovirus infection compared to the control group; *n* = 3, scale bar = 50 μm. (**D**) Representative images of transwell migration assay upon FGF12 adenovirus infection in ORS cells; *n* = 3, scale bar = 50 μm. (**E**) Wound scratch assay results were quantified and represented as percentages of open wound area; *n* = 3. (**F**) Quantification of transwell migration assay indicated that ORS cell migration increased at the MOI of 300 and 3000. All data expressed as mean ± standard error (* *p* < 0.05, ** *p* < 0.01, *** *p* < 0.001 versus the control group).

Since FGF12 knockdown has inhibited the proliferation and migration of ORS cells, it was hypothesized that FGF12 overexpression will promote the proliferation and migration properties in ORS cells, accelerating the hair growth cycle. First, to confirm the overexpression of FGF12 by adenovirus infection, qRT-PCR was carried out. The result showed a significant increase in FGF12 expression at the mRNA level (Figure 4A). Intriguingly, the upregulation of FGF12 in ORS cells did not show a dramatic effect on the proliferation rate (Figure 4B). Meanwhile, the result of the wound-scratch migration assay indicated that the overexpression of FGF12 in ORS cells promoted the cell migration at 300 and 300 multiplicities of infection (MOI) compared to the control group which was infected with LacZ adenovirus (Figure 4C,E). In addition, the migration property of FGF12 overexpressed ORS cells was evaluated by a transwell migration assay. The results exhibited a higher number of migrated ORS cells at 300 and 3000 MOI compared to the control group (Figure 4D,F).

### 2.5. FGF12 Upregulation in ORS Cells Promoted the Hair Growth in C3H/HeN Mouse

**Figure 5:**
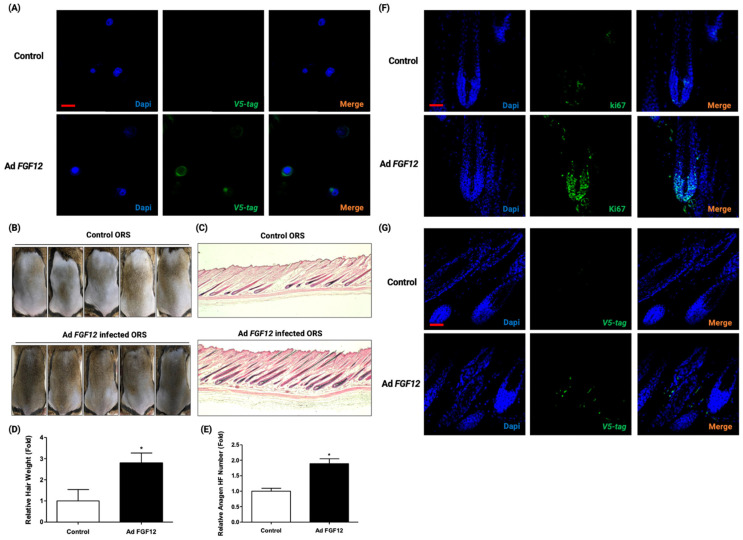
FGF12 overexpressing ORS cell injection in mouse dorsal skin at telogen phase promoted anagen induction in vivo. (**A**) Immunofluorescence staining with anti-V5-tag antibody in Adenovirus (Ad)-infected ORS cells confirmed that the virus infection was successfully carried out. Green indicates FGF12 and blue indicates DAPI. (**B**) The telogen-anagen transition was observed in C3H/HeN mouse. ORS cells were infected with LacZ Ad as control, and FGF12 Ad-infected ORS cells were used for the test group. Each group of Ad-infected ORS cells was injected into mouse dorsal skin and the images were taken after 14 days of injection; *n* = 5. (**C**) Hematoxylin and eosin (H&E) staining of the mouse dorsal skin in each group is shown. (**D**) The hair weight of each group was measured and quantified; *n* = 5. (**E**) Quantification of anagen follicle number from H&E staining indicated that overexpression of FGF12 in ORS cells promoted hair growth. (**F**) Immunofluorescence staining with the anti-ki67 antibody of mouse dorsal tissue samples harvested from telogen-to-anagen transition assay. (**G**) Immunofluorescence staining with the anti-V5-tag of mouse dorsal tissue samples harvested from telogen-to-anagen transition assay. All data expressed as mean ± standard error (* *p* < 0.05 versus the control group). scale bar = 50 μm.

Considering that the adenovirus (Ad)-mediated overexpression of FGF12 promoted the migration of ORS cells, it suggested the idea of hair-promoting effect by FGF12 upregulation. To observe the hair growth-promoting effect of FGF12 in ORS cells, FGF12 overexpressed ORS cells were injected into telogen phase mouse dorsal tissues. For the negative control group, Ad-LacZ infected ORS cells were injected. Before injection, immunofluorescence staining was performed to confirm whether Ad-FGF12 have infected successfully in ORS cells. As the Ad-FGF12 consisted of a V5-tag sequence, infection of Ad-FGF12 was confirmed by the detection of an anti-V5 antibody (Figure 5A). With FGF12 overexpressed ORS cells, an anagen induction experiment was carried out. Compared to the control group, the injection of FGF12 overexpressed ORS cells facilitated the telogen-anagen transition (Figure 5B). Hematoxylin and eosin (H&E) staining of the dorsal tissues showed a higher number of mature hair follicles in FGF12 overexpressed dorsal skin compared to the negative control group (Figure 5C,D). The hair weight was about 2-fold higher in FGF12 overexpressed ORS injected mice (Figure 5C). Immunostaining of anti-ki67 antibody was performed on the mouse dorsal skin samples harvested from the telogen-to-anagen transition assay. As a result, the expression of ki67 was higher in the FGF12 overexpressed group. This indicated that the proliferation of hair matrix cells had been triggered, in order to induce the anagen phase (Figure 5F). Additionally, to confirm how injected ORS affected the hair growth in vivo, we performed immunofluorescence staining of mouse dorsal tissue samples harvested from the telogen-anagen transition assay. As a result, we could observe that FGF12 overexpressed ORS cells were located near hair follicles, affecting neighboring ORS cells or other hair cells to migrate or proliferate during the anagen phase (Figure 5G).

### 2.6. Upregulation of Growth Factors in ORS Cells by Ad-Mediated FGF12 Overexpression

**Figure 6:**
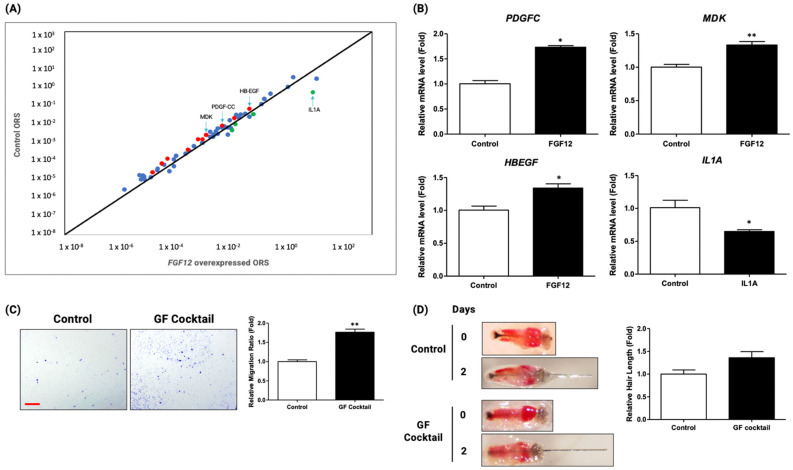
Overexpression of FGF12 in ORS cells upregulated various growth factors and exhibited the potential for a hair growth-promoting effect. (**A**) A scatter plot of RT2 profiler human growth factor PCR array of FGF12 overexpressed HORS cell is shown; *n* = 3. Red dots indicate genes that are related to hair growth and upregulated >2-fold. Green dots indicate genes that are related to hair loss. Blue dots include genes that are not related to hair growth. (**B**) Upregulation of various growth factors by FGF12 overexpression was confirmed through qRT-PCR; *n* = 3. (**C**) The growth factor cocktail was treated to ORS cells. The migration has increased by GF cocktail treatment and the quantification of the migration rate is shown; *n* = 3, scale bar = 50 μm. (**D**) Organ culture with GF treatment showed that hair fiber with growth factors slightly grew longer than the control group; x1.5 magnification. All data expressed as mean ± standard error (* *p* < 0.05, ** *p* < 0.01 versus the control group).

To find out how the FGF12 overexpression promotes migration and proliferation in ORS cells, a human growth factor array analysis was performed. ORS cells that are overexpressed with FGF12 Ad are used for the array and the growth factor array was carried out using qRT-PCR. The results are illustrated in the scatter plot (Figure 6A). Red dots in the scatter plot indicate the growth factors that are known to have a hair growth-promoting effect, whereas green dots indicate the growth factors that are known to possess a hair loss effect. The scatter plot showed that the expressions of several genes with hair growth-promoting effect have increased while a few number genes with hair loss effect have decreased. Among the red dots, platelet-derived growth factor C (PDGF-C), midkine (MDK), and heparin-binding epidermal growth factor-like growth factor (HB-EGF) increased their expression and the expression of IL1A (Green dot) have decreased (Figure 6A). Moreover, the expression changes of those growth factors were confirmed with other primers by qRT-PCR (Figure 6B). To confirm whether these growth factors are involved in hair growth promotion, the growth factor cocktail treatment was performed in ORS cells and mouse vibrissae follicles. Growth factor cocktail treatment in ORS cells promoted the migration rate in the transwell migration assay (Figure 6C). Additionally, mouse vibrissae follicles were treated with the growth factor cocktail. The result exhibited that the mouse vibrissae follicle cultured with the growth factor cocktail promoted the growth of hair follicle fiber length (Figure 6D). Altogether, the results demonstrated that FGF12 overexpression in ORS cells promotes hair growth by regulating the expression of various growth factors involved in the hair growth cycle.

## 3. Discussion

The role of many fibroblasts growth factor (FGF) family members has been studied in various research areas including hair follicle regeneration. As an example, FGF1 localized in inner root sheath cells and other hair cells promotes growth factor secretion to increase the proliferation of outer root sheath (ORS) cells [35]. FGF2 is localized in the hair matrix and ORS cells and exhibits a hair-growth promoting effect by increasing hair cell proliferation [21,48,49]. FGF2 increased the proliferation rate of dermal papilla (DP) cells and ORS cells [25]. Additionally, Osada et al. demonstrated mouse vibrissae hair follicle induction by subcutaneously injecting DP cells cultured with the medium containing FGF2 [50]. On the contrary, FGF5 induces the catagen phase during the hair cycle and decreases the hair length [29,51]. In the present study, we first demonstrated that FGF12 primarily exists in the ORS region and DP region. However, siRNA-mediated knockdown of DP cells did not show any effect (Figure 3B), while ORS cells exhibited hair-promoting effects. Hence, it is assumed that FGF12 might not show a dramatic hair-promoting effect. However, as ORS cells also play a key role during hair growth, this study investigated the effect of FGF12 on ORS cells during hair growth.

In this study, transfection of siRNA for FGF12 relayed the telogen-anagen transfection and decreased the vibrissae hair length. To confirm the hair growth induction by FGF12, we subcutaneously injected FGF12 adenovirus into the mouse dorsal skin after the depilation. However, the hair growth was not significantly induced by adenovirus injection. It is assumed that the direct injection of the adenovirus into the dorsal skin might not efficiently affect the target organ which is the hair follicle. Hence, we infected ORS cells with FGF12 adenovirus and injected FGF12-overexpressing ORS cells in mouse dorsal skin. Overexpression of FGF12 in ORS cells was confirmed with the immunofluorescence staining (Figure 5A). Compared to the control group, FGF12-overexpressed ORS cell injection showed a hair-growth promoting effect (Figure 5B,C). Accordingly, the results suggest that FGF12 is a hair-growth-promoting factor. Furthermore, to confirm how the injected ORS cells affected the hair growth in vivo, we performed immunofluorescence staining of mouse dorsal tissue samples harvested from the telogen-anagen transition assay. As a result, we could observe that FGF12 overexpressed ORS cells were located near hair follicles (Figure 5G). Hence, it could be assumed that ORS secretes several growth factors (PDGFC, HBEGF, MDK) affecting neighboring ORS cells or other hair cells to migrate or proliferate during the anagen phase.

From growth factor array results, we observed the upregulation of platelet-derived growth factor C (PDGF-C), Midkine (MDK), and heparin-binding epidermal growth factor-like growth factor (HB-EGF) by FGF12 overexpression, and the treatment of those growth factor cocktails to ORS cells showed the stimulation of ORS cells migration and the promotion of mouse vibrissae hair growth (Figure 6C,D). In addition, previous studies demonstrated the hair-promoting function of PDGF-C, MDK, and HB-EGF during the hair cycle by inducing the proliferation and migration of hair cells [44,52,53,54]. HB-EGF also is known to have the hair-growth promotion effect by inducing the growth and the migration of adipose-derived stem cells (ASCs) located near the hair follicles [54]. MDK is a popular growth factor involved in tissue regeneration and hair growth [55,56]. According to Figure 6, PDGF-C was one of the top three upregulated genes in ORS cells by FGF12 overexpression. PDGF members such as PDGF-A, -B, and -C are known as the hair-growth promoting factor [57]. PDGF-A and -B are known to induce and maintain the anagen phase during the hair cycle [58]. Furthermore, Choi et al. revealed that PDGF-C promotes DP proliferation, resulting in hair-growth promotion [52]. Altogether, our work demonstrated that hair growth was in part promoted via the upregulation of several growth factors (PDGF-C, MDK, HB-EGF) by FGF12.

A previous study by Nakayama et al. showed the suppression of radiation-induced apoptosis in mast cells by FGF12 overexpression. From the study, overexpression of FGF12 suppressed the apoptosis induced by MEK inhibitors suggesting that FGF12 regulated cell survival through the ERK pathway [59]. MEK/ERK pathway is known as a significant downstream effector of the Ras small GTPase which plays an essential role in cell proliferation and migration by growth factor regulation [60,61]. In a previous study, we found the growth factor secretion was primarily regulated by MEK/ERK pathway [52,62,63]. Although we did not further examine the mechanism of growth factor secretion, the previous research suggested that FGF12 might regulate the expression of growth factors by the MEK/ERK pathway. Therefore, we conducted ERK/MEK inhibition experiment on FGF12 overexpressing ORS cells. When the ERK/MEK inhibitor (PD98059) was used to treat FGF12 overexpressed ORS cells, the expression of growth factors (HB-EGF, PDGF-C) decreased (Supplement Figure S1).

Intriguingly, a recent study demonstrated FGF12 as an inhibitor of vascular smooth muscle (VSM) cell remodeling in pulmonary arterial hypertension [46]. In addition, Song at el. showed that FGF12 inhibited the VSM cell proliferation and promoted the differentiation of mouse embryonic stem cells and human dermal fibroblasts through PDGF-BB inhibition [64]. On the other hand, our study suggested a different function of FGF12 on ORS cells which is the stimulation of migration and proliferation of ORS cells with the hair-growth promoting effect (Figure 4). As an FGF family member, FGF12 might have one or more pathways resulting in various effects in different types of cells. However, other than the interaction with Islet-Brain-2 (IB2) binding, specific binding of FGF12 is not yet discovered which at the same time suggests the possibility of unidentified pathways such as binding to scaffold proteins [42]. Hence, further investigation on FGF12 is required for these questions.

FGF family has various biological functions and regulation of FGF12 in the hair growth cycle was investigated in this study. FGF12 is primarily expressed in ORS cells and is high during the anagen phase of hair follicles. Knockdown of FGF12 delayed telogen-to-anagen transition in mice and decreased the hair length in the vibrissae hair follicle. Though the hair growth promotion by FGF12 overexpression was not dramatically induced, the result showed that FGF12 overexpressed ORS cells induced the anagen phase from the telogen mouse model. In summary, FGF12 induced hair growth by stimulating the migration/proliferation of ORS cells with the regulation of several growth factors during the anagen phase. These results introduce FGF12 as a potential target in hair loss therapy.

## 4. Materials and Methods

### 4.1. Cell Culture

Human outer root sheath (HORS) cells at passages of 3 to 5 were grown in an EpiLife medium consisting of 60 μM calcium (Gibco, New York, NY, USA), 1% EpiLife Defined Growth Supplement (EDGS) (Gibco), and 1% penicillin-Streptomycin (Gibco). Human dermal papilla (HDP) cells at passages of 4 to 8 were cultured with follicle dermal papilla cell growth medium consisting of a supplement mix (PromoCell, Heidelberg, Germany) and 0.1% antibiotic-antimycotic (Gibco). Cell incubation was conducted at 37 °C in 5% CO_2_.

### 4.2. Small Interfering RNA (siRNA) Transfection

For the siRNA transfection in HDP and HORS cells, scramble siRNA or FGF12 siRNA transfection was performed with Lipofectamine RNAiMAX reagent (Invitrogen, Grand Island, NY, USA). Scramble siRNA was used for the control group. The siRNA transfection in vivo was carried out with in-vivo-jetPEI (Polyplus-Transfection, New York, NY, USA) reagent as a delivery agent.

### 4.3. Adenovirus Infection

Adenovirus infection was used to overexpress FGF12 in cells and for the control group, LacZ adenovirus was used. A vector expressing human FGF12 cDNA was purchased from Origene (Rockville, MD, USA). For the recombinant adenovirus plasmid construction, the pShuttle-CMV vector was used for the human FGF12 cDNA subcloning. Recombinant adenovirus was constructed and purified by ViraQuest Inc. (North Liberty, IA, USA).

### 4.4. Immunofluorescence Staining

For the Immunofluorescence staining of mouse dorsal tissue, sample tissue embedded in paraffin block was sectioned using a microtome (Microm HM340E, Thermo Scientific, Waltham, MA, USA) longitudinally. HORS cells and tissue sections were stained with anti-FGF12 antibody (Abcam, Cambridge, UK; 1:200) and anti-V5 antibody (Cell signaling; 1:200) overnight. Alexa Fluor 488 goat anti-rabbit IgG was used as a secondary antibody and nuclei of cells were stained with 4′6-diamidino-2-phenylindole (DAPI). All images of immunofluorescence staining were visualized using a Zeiss LSM700 confocal microscope (Carl Zeiss, Jena, Germany).

### 4.5. Telogen-to-Anagen Transition Assay in C3H/HeJ mice

The dorsal skin of male C3H/HeJ mice was shaved with an electric shaver at 7-weeks of age when all the hair follicles are in the telogen phase. To ensure that non-pigmented dorsal skin was clearly exposed, the remaining hair was removed by depilatory cream. For FGF12 knockdown, 6 μg of scrambled siRNA (Control) or FGF12 siRNA was subcutaneously injected into the dorsal skin of mice every 72 h for 13 days. For FGF12 overexpression, FGF12 overexpressing ORS cells were directly injected into the dorsal skin of mice subcutaneously and for the control group, LacZ infected ORS cells were injected. Before the injection, ORS cells were first cultured for 24 h after the LacZ adenovirus (Control) or FGF12 adenovirus infection. Then, 5 × 10^4^ ORS cells were harvested and injected every once a week for 14 days. At 13–14 days, mice were sacrificed for the hair and dorsal tissue obtaining. Telogen to anagen transition rate was demonstrated by the quantification of the number of anagen follicles, hair weight, and darkness of dorsal skin due to hair regrowth.

### 4.6. Hematoxylin and Eosin (H&E) Staining

The harvested dorsal skin samples were embedded in paraffin blocks. Paraffin block samples were cut using a microtome (Microm HM340E, Thermo scientific) longitudinally. Those paraffin section slides were dewaxed for 30 min with xylene and dehydrated in 100%, 90%, 80%, and 70% ethanol for 2 min each. Sample slides were then stained with Mayer’s hematoxylin (Sigma-Aldrich Corporation, Saint Louis, MO, USA) for 10 min. After that, sample slides were washed three times and stained with Eosin Y (Sigma-Aldrich) subsequently for 90 s. They were dehydrated again with 70%, 80%, 90%, and 100% ethanol for 2 min and washed in xylene for 20 min. Finally, H&E-stained sample tissues were visualized by adding a mounting solution (toluene solution un1294, Thermochem Inc, Santa Rosa, CA, USA).

### 4.7. Hair Follicle Organ Culture

A hair follicle organ culture experiment was carried out using isolated vibrissae follicles of 4-week-old female C57BL/6J at the anagen phase. Images of the isolated hair follicles were taken. Isolated hair follicles were transfected with 6 μg of scramble (Control) or FGF12 siRNA and incubated in William’s E medium containing 10 μg/mL insulin, 2 mM L-glutamine, and 10 ng/mL hydrocortisone. After 48 h of incubation, the length of the hair was imaged using a ZEISS Observer D1 microscope (Carl Zeiss). The change in hair length from 0 h to 48 h was analyzed using ImageJ software (version 1.45; National Institutes of Health, Bethesda, MD, USA).

### 4.8. Western Blot

Protein was extracted from the HORS cell for the western blot. For the protein quantification, BCA analysis was carried out using a BCA analysis kit (Pierce BCA protein assay kit; Thermo Fisher Scientific, Waltham, MA, USA). During gel running, 25 μg of proteins were run on a 10% acrylamide gel. Proteins were then transfer to polyvinylidene fluoride membrane (Immobilon-P; Merck Millipore, Darmstadt, Germany). Blocking of the membrane was done by using 5% bovine serum albumin and incubated with anti-FGF12 antibody and anti-alpha tubulin antibody (Housekeeping gene) overnight. The membrane was washed with Tris-buffered saline containing Tween-20 for 10 min, 3 times. After that, the membrane was incubated for one hour with a secondary antibody which was a peroxidase-labeled anti-mouse antibody and a peroxidase-labeled anti-rabbit antibody. The membrane was washed with TBS-T again and imaged with Image Quant LAS 4000 (GE Health care Life Science, Bensalem, PA, USA) after the reaction with a chemiluminescent solution (Immobilon Western, Millipore, Burlington, MA, USA).

### 4.9. Real-Time Quantitative Reverse Transcription-Polymerase Chain Reaction (qRT-PCR)

Total RNA from siRNA transfected and adenovirus infected HORS cells and mouse dorsal skin samples was extracted using TRIzol Reagent (Thermo Fisher Scientific). Extracted RNA samples were reverse transcribed using HelixCript Thermo Reverse Transcription System (NanoHelix Co., Ltd., Daejeon, Korea) following the manufacturer’s instructions. The fold changes of gene expressions were normalized by the housekeeping gene (GAPDH) based on the ΔΔCT method [65]. Primer sequences used in this research are shown in Table 2.

### 4.10. Proliferation Assay

HORS and HDP cells were seeded in 6-well plates with the number 1.5 × 10^4^ per well. Afterward, siRNA transfection or adenovirus infection was carried out and incubated for 72 h. For the siRNA transfection, scramble or FGF12 siRNA was treated dose-dependently (1, 10, 100 nM) and for the adenovirus infection, LacZ adenovirus or FGF12 adenovirus was treated dose-dependently (300, 3000 MOI) in the medium. Cell proliferation was determined by a Cell-Counting Kit-8 assay (Dojindo Molecular Technologies, Inc., Rockville, MD, USA).

### 4.11. Scratch Migration Assay

HORS cells were seeded in 6-well plates and cultured until 100% confluency was reached in every well. The wound scratch was then created using a sterile 1000 μL pipette tip and floating cell debris was removed by washing them with phosphate-buffered saline (PBS) 3 times. Subsequently, cells were transfected with siRNA or infected with adenovirus. For the siRNA transfection, scramble or FGF12 siRNA was treated dose-dependently (1, 10, 100 nM) and for the FGF12 overexpression, LacZ (Control) or FGF12 adenovirus was infected does-dependently (300, 3000 MOI) to the ORS cell. After 48 h of incubation, the image of the migration results was acquired by a ZEISS Observer D1 microscope (Carl Zeiss).

### 4.12. Transwell Migration Assay

First, the upper transwell chamber was coated with fibronectin. Then, siRNA transfected or adenovirus infected HORS cells were seeded into the transwell chamber with a density of 1.5 × 10^4^ cells per well. For the siRNA transfection, scramble or FGF12 siRNA was treated dose-dependently (1, 10, 100 nM) and for the FGF12 overexpression, LacZ (Control) or FGF12 adenovirus was infected dose-dependently (300, 3000 MOI) to the ORS cell. After 48 h, cells on the top surface of the transwell chamber were washed with PBS 3 times. The fixation and staining of migrated cells on the lower surface of the transwell chamber were performed by incubating them with crystal violet dye-containing 4% paraformaldehyde for 30 min. The migration of HORS cells was imaged and the relative number of migrated cells was obtained.

### 4.13. Human Growth Factor Array Analysis

The expression changes of growth factor genes in FGF12 overexpressed HORS were determined using an RT2 PCR array kit (PAHS-041Z; Qiagen, Hilden, Germany), and the fold changes in the gene expression were analyzed by the RT2 PCR array data analysis web portal. FGF12 overexpression of ORS cells was carried out using FGF12 adenovirus.

### 4.14. Growth Factor Cocktail Treatment

The growth factor cocktail consists of recombinant human platelet-derived growth factor-CC (PDGF-CC; PeproTech, Rocky Hill, NJ, USA), recombinant heparin-binding epidermal growth factor-like growth factor (HB-EGF; PeproTech), and human recombinant Midkine (MDK; PeproTech).

### 4.15. Statistical Analysis

All data are demonstrated as the mean ± standard deviation of at least three independent experiments. For the analysis between two groups, Student’s t-test was used. When more than two groups are compared, a one-way analysis of variance (ANOVA) test followed by Dunnett’s post hoc test was used. The statistical significance was set at a p-value of less than 0.05. GraphPad Prism 5.01 (GraphPad Software Inc., San Diego, CA, USA) was used for all statistical analysis.

## Figures and Tables

**Table 1:** Effects of FGFs in hair growth (+/−).

Gene	Effect	Function	References
*FGF1*	+	Involved in HF differentiation, prevents radiation-induced apoptosis in HF	[21,22,23]
*FGF2*	+	Involved in the proliferation of HF cells, elongation of the anagen phase	[24,25,26]
*FGF5*	−	Catagen induction, blocks DPC activation during anagen	[27,28,29]
*FGF7*	+	Elongation of the anagen phase, hair germ proliferation, stem cell activation	[3,30]
*FGF8*	−	Inhibits proliferation of epidermal cells	[31]
*FGF9*	+	Induces HF neogenesis after wounding	[32]
*FGF10*	+	HF formation and morphogenesis	[33,34]
*FGF13*	+	Regulates bulge/ORS cells, reduced expression in hypertrichosis	[35,36]
*FGF18*	−	Telogen induction, maintains stem cell quiescence	[37,38]
*FGF20*	+	Formation of dermal condensations, placode development	[39,40]
*FGF21*	+	Secondary HF development	[41]

**Table 2:** Primer sequences used in the qRT-PCR analysis.

Gene	Forward (5′-3′)	Reverse (5′-3′)
*GAPDH*	GGAGCGAGATCCCTCCAAAAT	GGCTGTTGTCATACTTCTCATGG
*FGF12*	GGGACCAAGGACGAAAACAG	TTGCTGGCGGTACAGTGTG
*PDGFC*	TGAACCAGGGTTCTGCATCCAC	TAAGCAGGTCCAGTGGCAAAGC
*MDK*	CGCGGTCGCCAAAAAGAAAG	TACTTGCAGTCGGCTCCAAAC
*HB-EGF*	TGTATCCACGGACCAGCTGCTA	TGCTCCTCCTTGTTTGGTGTGG
*IL1A*	TGTATGTGACTGCCCAAGATGAAG	AGAGGAGGTTGGTCTCACTACC

## Data Availability

All data supporting the findings of this study are available within the paper published online.

## Supplementary Materials

The following supporting information can be downloaded at: https://www.mdpi.com/1422-0067/23/16/9467/s1.

## Abbreviations

FGF	Fibroblast growth factor
HORS	Human outer root sheath
H&E	Hematoxylin and eosin
HDP	Human dermal papilla
MOI	Multiplicity of infection
Ad	Adenovirus
GF	Growth factor
PDGF-CC	Platelet-derived growth factor-CC
HB-EGF	Heparin-binding epidermal growth factor-like growth factor
MDK	Midkine
VSM	Vascular smooth muscle
IB2	Islet-Brain-2
