# Supplementary material for: Hair Growth Regulation by Fibroblast Growth Factor 12 (FGF12)
Source: Int J Mol Sci. 2022 Aug 22;23(16):9467. doi: 10.3390/ijms23169467 (PMC9409131; doi:10.3390/ijms23169467)
Supplement: Supplementary file 1 [file ijms-23-09467-s001.zip › ijms-1787124-supplementary.pdf]

Supplementary Data

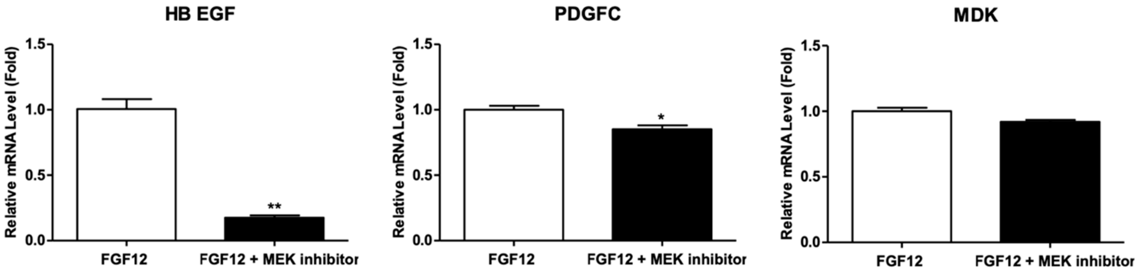

Figure S1. ERK/MEK inhibition study using PD98059. The expression of HB-EGF and PDGFC decreased with the PD98059 treatment.
